# Discrete elemental analysis on the effect of particle morphology and size on interparticle contact force

**DOI:** 10.1371/journal.pone.0337345

**Published:** 2025-11-25

**Authors:** Yongfeng Zhu, Wei Xiong, Wen Fan

**Affiliations:** 1 School of Geology Engineering and Geomatics, Chang’an University, Xi’an, Shaanxi, China; 2 Key Laboratory of Western China’s Mineral Resources and Geological Engineering, Ministry of Education, Xi’an, Shaanxi, China; 3 Xinjiang Hetian College, Hetian, China; 4 China Electronic Research Institute of Engineering Investigations and Design, Xi’an, Shaanxi, China; Graphic Era Deemed to be University, INDIA

## Abstract

Particle morphology and size are fundamental characteristics that significantly influence the mechanical behavior of granular materials. This study introduces key parameters—aspect ratio (Ω), sphericity (S), and equivalent diameter (Dₑ)—into a modified Hertz-based contact model to conduct a multiscale study using contact mechanics theory and the discrete element method (DEM). A series of two-particle tests and triaxial compression simulations were performed. The results show strong agreement between numerical simulations and theoretical predictions at the particle scale, validating the modified contact model. At the sample scale, the peak deviatoric stress increased by approximately 15–40% as aspect ratio decreased from 1.00 to 0.33 and sphericity decreased from 1.00 to 0.11. Similarly, increasing the equivalent diameter from 3.78 mm to 8.82 mm led to a 20–35% rise in peak stress. At the particle scale, both normal and tangential contact forces increased with larger equivalent diameters but exhibited complex dependencies on morphology due to varied contact patterns. These findings enhance the understanding of how particle-scale characteristics influence macroscopic mechanical properties.

## 1. Introduction

Particle morphology and size are fundamental characteristics of granular materials, such as soils and sands, that profoundly influence their macroscopic mechanical behavior [1,2]. These characteristics are not merely geometric descriptors but are intrinsically linked to the material’s formation history and subsequent physicochemical actions, ultimately governing properties like shear strength, compaction, and permeability [1–4]. Consequently, a deep understanding of the role played by particle morphology and size is crucial for advancing the design and construction practices in geotechnical and particulate engineering.

Numerous experimental tests have been conducted to quantify the impact of particle characteristics on mechanical behavior [5–11]. Traditional laboratory tests on natural materials [5–7,10,11] and innovative approaches using 3D-printed particles [12–17] have provided valuable insights. For instance, 3D printing has enabled the systematic exploration of morphology effects on macro-mechanical properties [12–14], packing density [15], and particle crushability [16,17]. While these experimental efforts have successfully established important correlations between particle properties and macroscopic response [18,19], they are inherently limited to observing bulk phenomena. They cannot readily access critical microscopic information, such as interparticle contact forces and fabric evolution, which are the fundamental drivers of the observed macroscopic behavior.

The discrete element method (DEM) proposed by Cundall and Strack [20], is an important tool to overcome these limitations. DEM facilitates the investigation of both macroscopic and microscopic mechanical properties by explicitly modeling the interaction of individual particles. Many researchers have employed DEM to study the influence of morphological parameters (e.g., aspect ratio, angularity, sphericity) and particle size distribution on the behavior of granular assemblies [21–25]. However, a review of the literature reveals a significant focus on the sample-scale macroscopic effects, often neglecting a detailed and mechanistic analysis of the particle-scale contact forces. While some recent studies have begun to use machine learning to accelerate DEM computations and map complex micro-macro relationships [26–28], a comprehensive understanding of how morphology and size directly govern interparticle contact mechanics—the very origin of granular behavior—remains underdeveloped. This constitutes a critical knowledge gap.

Therefore, this study aims to bridge this gap by conducting a rigorous multiscale investigation (spanning the particle, contact, and sample scales) into the effects of particle morphology and size on mechanical characteristics. Unlike previous studies, we integrate key morphological parameters (aspect ratio and sphericity) and the size parameter (equivalent diameter) directly into a modified Hertz-Mindlin contact model based on contact mechanics theory, derive and present the modified theoretical framework. Subsequently, we validate the model through a series of controlled two-particle tests. Finally, we employ DEM simulations of triaxial compression tests to explore the consequences of these particle-scale effects on sample-scale strength, contact force distribution, and fabric anisotropy. This integrated approach provides a comprehensive understanding that can offer robust theoretical support for optimizing engineering practices involving granular materials.

## 2. Theoretical framework

In order to explore the effect of particle characteristics on interparticle contact force, the particle morphology and size parameters such as aspect ratio, sphericity and equivalent diameter were introduced into the analysis of contact forces based on contact mechanics, carrying out the theoretical analysis of normal and tangential contact force between non-spherical particles at the particle scale. This section details the development of the modified Hertz-Mindlin contact model, which incorporates particle morphology and size. The core idea is to express the key geometric parameter in contact mechanics—the mean effective radius, *R*ₘ—as a function of the fundamental particle characteristics: aspect ratio (*Ω*), sphericity (*S*), and equivalent diameter (*Dₑ*).

### 2.1. Particle morphology and size parameters

In accordance with previous studies [21,22], particle morphology and size parameters including aspect ratio, sphericity, and equivalent diameter are adopted. To quantitatively describe non-spherical particles, we adopt a triaxial ellipsoid model, and can be mathematically defined by Eq (1) (Note that a, b, c are the short, middle, and long semi-axes of the ellipsoid, respectively. For simplicity of calculation, the short and middle semi-axes of the ellipsoid were set to be the same value.):


$$\frac{x^{\text{2}}}{a^{\text{2}}}+\frac{y^{\text{2}}}{b^{\text{2}}}+\frac{z^{\text{2}}}{c^{\text{2}}}=\text{1}(a=b\leq c)$$ (1)

Aspect ratio (Ω) of the ellipsoid, representing the ratio of the minimum ferrite diameter (*W*) to the maximum ferrite diameter (*L*), is calculated by Eq (2):


$$\Omega =\frac{W}{L}=\frac{a}{c}$$ (2)

Sphericity (*S*) of the ellipsoid is defined as the ratio of the surface area of a sphere with the same volume as the ellipsoid particle to the surface area of the ellipsoid particle, as calculated by the following Eq (3) [29,30]:


$$S=\frac{A_{sp}}{A}=\frac{\sqrt[{\text{3}}]{\text{3}\text{6}\pi V^{\text{2}}}}{A}=\frac{ab}{c^{\text{2}}}$$ (3)

where *V* is the volume of the ellipsoid particle, *A* is the surface area of the ellipsoid particle, $A_{sp}$ is the surface area of a sphere with the same volume as the particle. Due to the *a = b* assumption, *S* = *Ω*^*2*^, which makes theoretical framework incorporate 2 independent parameters (morphological: *Ω*; size: *D*_*e*_). However, the assumption that *a *=* b* adopted in the article is merely to simplify calculations. In reality, a is not equal to b, so it is still necessary to treat *S* and *Ω* as two independent parameters.

Equivalent diameter ($D_{e})$ of the ellipsoid, which means the diameter of a sphere with the same volume, is given by Eq (4):


$${D_{e}}^{\text{3}}=\frac{\text{6}V}{\pi }=\text{8}abc$$ (4)

The parameters *Ω*, *S*, and *Dₑ* provide a complete description of the particle’s form and size. Then the short, middle, and long semi-axes of an ellipsoid can be expressed in terms of the morphological parameters of the ellipsoidal particles in Eq (5):


$$\left\{ \;\;\;\begin{array}{c} a=a\left(\Omega ,S,D_{e}\right)=\Omega {\left(\frac{{D_{e}}^{\text{3}}}{\text{8}S}\right)}^{\frac{\text{1}}{\text{3}}}\\ b=b\left(\Omega ,S,D_{e}\right)=\frac{S}{\Omega }{\left(\frac{{D_{e}}^{\text{3}}}{\text{8}S}\right)}^{\frac{\text{1}}{\text{3}}}\\ c=c\left(\Omega ,S,D_{e}\right)={\left(\frac{{D_{e}}^{\text{3}}}{\text{8}S}\right)}^{\frac{\text{1}}{\text{3}}} \end{array}\right.$$ (5)

Using Eq (5), the geometric dimensions of any ellipsoidal particle in the simulation can be uniquely determined from these input parameters. The regions of deformation on the contact surfaces are quite small and can be approximated as quadric curved surfaces, because most contact problems are localized [31,32]. In other words, the properties of the contact are primarily influenced by the local geometries in the immediate area of the contact point, which are distinguished by two principal radii of curvature. According to the Appendix A which is derived from references [33,34], the principal radii of curvatures $R_{c}$ can be described by particle morphology and size parameters like the following Eq (6).


$$R_{c}=R_{c}(\Omega ,S,D_{e},\theta )$$ (6)

This equation is the pivotal step in our model modification. It allows us to dynamically calculate the effective contact geometry for any contact configuration between two non-spherical particles, rather than assuming a fixed radius.

### 2.2. Normal contact force

#### 2.2.1. Hertz normal contact force.

The central modification to the classical Hertz theory lies in bridging the macro-scale particle morphology with the micro-scale geometry at the contact point. Classical Hertz-Mindlin theory for spheres uses the constant particle radius. For non-spherical particles, the local geometry is characterized by the principal radii of curvature, *Rc*, at the contact point, which varies with the location and orientation of contact.

In the DEM, the motion of a single particle is calculated by using Newton’s second law of motion. The governing equation of the translation can be expressed as Eq (7):


$$m\dot{v}=F$$ (7)

where *m* is the mass of a non-spherical particle, $\dot{v}$ is the acceleration of the non-spherical particle and F is the contact force. The contact force includes elastic contact force and damping force in normal and tangential directions, which is defined as Eq (8):


$$F=F^{n}+F^{s}=F_{H}^{n}+F_{d}^{n}+F_{MD}^{s}+F_{d}^{s}$$ (8)

where $F^{n}$ is the normal contact force, $F^{s}$ is the shear contact force, $F_{H}^{n}$ is the elastic Hertz contact force in normal direction, $F_{d}^{n}$ is the viscous contact force in normal direction, $F_{MD}^{s}$ is the elastic contact in tangential direction, $F_{d}^{s}$ is the viscous contact force in tangential direction. The definitions and models of all forces mentioned will be described in the following sections.

**Fig 1:**
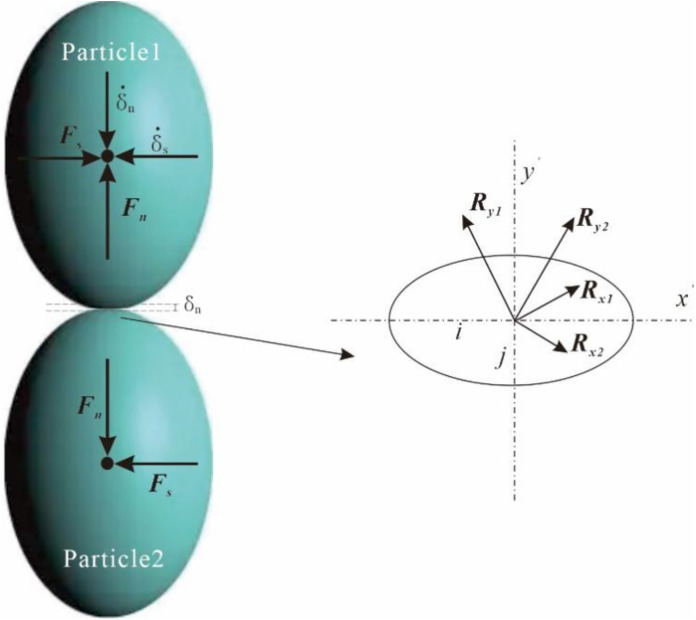
Two ellipsoidal particles in contact.

In this study, the theory of Hertz is employed to study the normal contact force, in which the Particle 1 is pressed to the Particle 2 along the normal direction (z axis in Fig 1), with a normal displacement $\delta_{n}$ and normal velocity ${\dot{\delta }}_{n}$. There is an elliptical area with major and minor semi-axes being *i* and *j* occurring when two ellipsoids contact with each other, as shown in Fig 1.

Pressure distribution in this elliptical area can be represented as Eqs (9–12) [34]:


$$p=p_{\text{0}}{\left(\text{1}-\frac{x^{\text{2}}}{i^{\text{2}}}-\frac{y^{\text{2}}}{j^{\text{2}}}\right)}^{\frac{\text{1}}{\text{2}}}$$ (9)

$$F=\int_{\text{0}}^{j}\int_{\text{0}}^{i}p(x,y)\left[\text{2}\pi y+\text{4}(x-y)\right]dxdy=\frac{\text{2}}{\text{3}}p_{\text{0}}\pi ij$$ (10)

$$ij=R_{m}\delta_{n}$$ (11)

$$p_{\text{0}}=\frac{\text{2}}{\pi }E_{e}{\left(\frac{\delta_{n}}{R_{m}}\right)}^{\text{1}/\text{2}}$$ (12)

where $\delta_{n}$ is the relative displacement between two particles, $R_{m}$ is the mean effective radius, as described in Eq (13):


$$\frac{\text{1}}{R_{m}}=\frac{\text{1}}{\text{2}}(\frac{\text{1}}{R_{\text{1}}}+\frac{\text{1}}{R_{\text{2}}})=\frac{\text{1}}{\text{4}}(\frac{\text{1}}{R_{x\text{1}}}+\frac{\text{1}}{R_{x\text{2}}}+\frac{\text{1}}{R_{y\text{1}}}+\frac{\text{1}}{R_{y\text{2}}})$$ (13)

where $R_{x}$ and $R_{y}$ are the effective radii of curvature in principal x and y direction; subscripts 1 and 2 indicate ellipsoid 1 and ellipsoid 2, respectively.

Equation 9 (pressure distribution) and Equation 13 (definition of *R*_*m*_) are foundational assumptions, while Equations 10–12 are direct consequences of these assumptions, derived via integration, geometric compatibility, or material constitutive relationships.

The effective modulus of elasticity $E_{e}$ is given by Eq (14):


$$\frac{\text{1}}{E_{e}}=\frac{\text{1}-\upsilon_{\text{1}}^{\text{2}}}{E_{\text{1}}}+\frac{\text{1}-\upsilon_{\text{2}}^{\text{2}}}{E_{\text{2}}}$$ (14)

where E and υ are respectively Young’s modulus and the Poisson ratio, subscripts 1 and 2 indicate body 1 and body 2, respectively. The Equation 14 is a well-established result in contact mechanics derived under the key assumptions: linear elasticity, isotropy, small deformations. Under these assumptions, the effective elastic modulus *E*_*e*_ is derived to characterize the combined stiffness of two contacting elastic bodies. The derivation involves solving the boundary value problem for the contact of two elastic half-spaces, using the principle of superposition to combine the contributions of each material’s elastic properties.

The normal contact force according to the Hertz theory $F_{H}^{n}$ can be given by the following Eq (15) [35].


$$F_{H}^{n}=F_{H}^{n}\left(\Omega ,S,D_{e}\right)=\frac{\text{4}}{\text{3}}E_{e}{R_{m}}^{\text{0}.\text{5}}{\left(\delta_{n}\right)}^{\text{1}.\text{5}}$$ (15)

#### 2.2.2. Dashpot normal contact force.

According to previous studies [36], the normal contact damping force is proportional to the relative normal velocity between the particles, as Eq (16),


$$F_{d}^{n}=C^{n}{\dot{\delta }}_{n}$$ (16)

where $C^{n}$ is the normal damping coefficient, proportional to the normal contact stiffness, given by Eq (17):


$$C^{n}=\text{2}\beta_{n}\sqrt{{m^{*}k}_{n}}$$ (17)

where $\beta_{n}$ is the normal damping ratio, $m^{*}$ is the effective mass, $k_{n}$ is the normal contact stiffness. We obtained the following Eq (18):


$$m^{*}=\frac{m_{\text{1}}m_{\text{2}}}{m_{\text{1}}+m_{\text{2}}},k_{n}=\frac{dF_{H}^{n}}{d\delta_{n}}=E_{e}\sqrt{\text{2}R_{m}\delta_{n}}$$ (18)

The normal viscous force is obtained as the following Eq (19):


$$F_{d}^{n}=F_{d}^{n}\left(\Omega ,S,D_{e}\right)=\text{2}\beta_{n}\sqrt{{m^{*}k}_{n}}{\dot{\delta }}_{n}$$ (19)

### 2.3. Shear contact force

#### 2.3.1. Mindlin–Deresiewicz shear contact force.

The tangential force is based on the work of Mindlin-Deresiewicz [37]. The tangential force follows Coulomb’s friction law and is proportional to shear displacement at each contact point. It is known that, during the initial stage of tangential deflection, only the outer annulus of the contact area experiences sliding between the two surfaces in contact, whereas the inner circle remains stationary. The tangential force exerted at this stage is lower than the frictional limit, $\mu F^{n}$, and varies nonlinearly with the tangential displacement. This phenomenon is commonly referred to as “partial slip” or “micro-slip,” and has been adequately addressed independently by Mindlin [38], in the context of elastic spheres subjected to a constant load.

The tangential stiffness is related to normal force, as given in Eq (20) [37].


$$\Delta F_{MD}^{S}=k_{s}\Delta \delta_{s}$$ (20)

where $\Delta F_{MD}^{S}$ is the relative shear force increment, $\Delta \delta_{s}$ is the relative shear displacement increment, $k_{s}$ is the tangential stiffness, described in Eq (21):


$$k_{s}=\frac{\text{2}G_{e}^{\text{2}/\text{3}}}{\text{2}-\nu_{e}}{\left[\text{3}(\text{1}-\upsilon_{e})R_{m}F^{n}\right]}^{\text{1}/\text{3}}$$ (21)

where $G_{e}$ is the effective shear modulus, $\nu_{e}$ is the effective Poisson’s ratio, $R_{m}$ is the mean effective radius, $F^{n}$ is the normal contact force. We obtained the following Eq (22):


$$G_{e}=\frac{\text{1}}{\text{2}}(G_{\text{1}}+G_{\text{2}}),\upsilon_{e}=\frac{\text{1}}{\text{2}}(\upsilon_{\text{1}}+\upsilon_{\text{2}})$$ (22)

where G and υ are respectively shear modulus and the Poisson ratio, subscripts 1 and 2 indicate body 1 and body 2, respectively.

When $\delta_{s}$ is larger than $\delta_{s,max}$, relative slip between particles then occurs. The tangential contact force by Mindlin–Deresiewicz theory is calculated as the following Eq (23) [6]:


$$F_{MD}^{s}=F_{MD}^{s}\left(\Omega ,S,D_{e}\right)=\left\{ \;\;\;\begin{array}{c} \frac{\text{2}G_{e}^{\text{2}/\text{3}}}{\text{2}-\nu_{e}}{\left[\text{3}(\text{1}-\upsilon_{e})R_{m}F^{n}\right]}^{\text{1}/\text{3}}\delta_{s}\left|\delta_{s}\right|\leq \delta_{s,max}\mathrm{\backslash vspace}1mm\\ \mu F^{n}\;\left|\delta_{s}\right|>\delta_{s,max} \end{array}\right.$$ (23)

#### 2.3.2. Dashpot tangential contact force.

In accordance with previous studies [36], the tangential contact damping is also proportional to the relative velocity between the particles, given by Eq (24):


$$F_{d}^{s}=C^{s}{\dot{\delta }}_{s}$$ (24)

where $C^{s}$ is the tangential damping coefficient, proportional to the tangential contact stiffness, described in Eq (25):


$$C^{s}=\text{2}\beta_{s}\sqrt{{m^{*}k}_{s}}$$ (25)

where $\beta_{s}$ is the shear damping ratio, $m^{*}$ is the effective mass, $k_{s}$ is the shear contact stiffness.

The tangential viscous force is obtained as the following Eq (26):


$$F_{d}^{s}=F_{d}^{s}\left(\Omega ,S,D_{e}\right)=\text{2}\beta_{s}\sqrt{{m^{*}k}_{s}}{\dot{\delta }}_{s}=\frac{\text{2}\beta_{s}G_{e}^{\text{1}/\text{3}}\sqrt{{\text{2}m}^{*}}}{\sqrt{\text{2}-\nu_{e}}}{\left[\text{3}(\text{1}-\nu_{e})R_{m}F^{n}\right]}^{\text{1}/\text{6}}{\dot{\delta }}_{s}$$ (26)

## 3. Validation of the theoretical model

### 3.1. Numerical model description

To validate the proposed theoretical framework, a modified Hertz contact model was implemented and integrated into the discrete element software PFC5.0 (Version 5.00.35). The force-displacement constitutive relationship and contact relationship between non-spherical particles were encoded in C++ within the user-defined contact model section. Leveraging the MCVS2010 and QT open-source development framework, this process was facilitated. Subsequently, the modified Hertz contact model was compiled into a dynamic link library (DLL) file using the debugging platform of MSVS2010. This DLL file was then loaded into PFC5.0 for numerical simulation to validate the model’s efficacy. Furthermore, a series of two-particles tests were conducted to ascertain whether the DLL file of the modified Hertz contact model can be successfully utilized in the PFC3D for numerical simulation.

In each DEM timestep, when a contact is detected between two ellipsoids, the contact point and orientation are determined. Based on this, the local *Rc* for each particle is computed via Eq (6), *R*ₘ is calculated via Eq (13), and finally, the contact forces are updated using the equations in Section 2.3. This process ensures that the mechanical behavior at every contact is consistent with the local geometric conditions defined by the particles’ shapes.

The DEM parameters were selected to align closely with the theoretical assumptions. For simplicity and better variable control, only head-on contacts between two ellipsoids were considered, as shown in Fig 1. This facilitates the control of variables such as principal radii of curvature, contact angle, and contact point. Both the normal and tangential relative displacements between ellipsoidal particles were manually set, with PFC’s implicit solver employed for the DEM analysis to ensure computational accuracy. Ellipsoidal particles were utilized in this research, with their short and middle semi-axes set to equal length (a = b). By changing the length of the long semi-axis (c), different prolate spheroids (c > b) were obtained, resulting in different aspect ratios (*Ω* = 1.00, 0.67, 0.50, 0.40, 0.33) and sphericities (*S*=1.00, 0.44, 0.25, 0.16, 0.11) to study the effect of particle morphology on normal and tangential contact force. Subsequently, the bubble packing algorithm [39] implemented in PFC3D to was employed to model these prolate spheroids by overlapping spheres called as clump, also mentioned in previous studies [40–41]. This method approximates the overall geometry of ellipsoids by distributing smaller spheres along the ellipsoidal contour, ensuring that the clump’s volume, centroid, and principal axes align with those of the target ellipsoid. This approximation is reasonably accurate for several reasons. On the one hand, the clump model effectively captures the key geometric features (e.g., curvature and aspect ratio) that govern contact mechanics. On the other hand, the clump’s overlapping spheres minimize artificial discontinuities in surface geometry.

**Fig 2:**
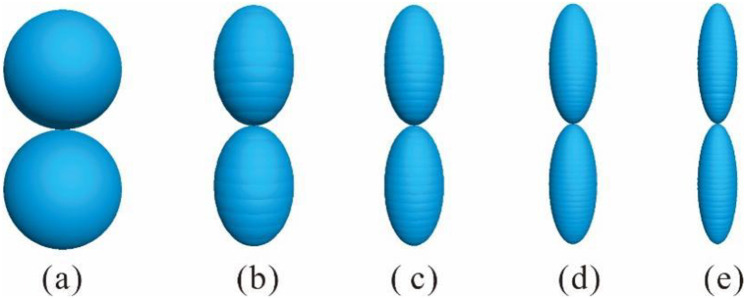
Different ellipsoidal particles with De = 6.30 mm used in the discrete element simulations. (a) P1: Ω = 1.00, S=1.00; (b) P2: Ω = 0.67, S=0.44; (c) P3: Ω = 0.50, S=0.25; (d) P4: Ω = 0.40, S=0.16; (e) P5: Ω = 0.33, S=0.11.

These particles, named P1, P2, P3, P4, and P5, respectively, are shown in Fig 2. These five particles were also set to have the same equivalent diameter. Meanwhile, different equivalent diameters (*D*_*e*_ = 3.78 mm, 5.04 mm, 6.30 mm, 7.56 mm, 8.82 mm) were assigned to ellipsoids (P3) to explore the effect of particle size on normal and tangential contact forces. These particles were named S1, S2, S3, S4, and S5, respectively. The material properties used in the DEM are listed in Table 1. These parameters were determined based on a combination of theoretical consistency and references to established literature [18,22,35,36]. The particles move according to the procedure outlined in the Section 2. The contact between two ellipsoids is active when the surface gap between these two ellipsoids is less than or equal to zero. In order to generate a normal contact, a small downward displacement of 5 μm in the normal direction was applied to the upper ellipsoid whose normal velocity is 1m/s while the lower ellipsoid remained fixed [31,35]. Friction was not considered in this stage, which complies with the Hertz theory. The tangential contact force was modeled based on the normal contact force. The normal displacement was maintained while a tangential displacement of 0.5 μm was slowly applied to the upper ellipsoid along the contact plane at a rate of 0.5 m/s [31,35]. Except for these displacements, all other degrees of freedoms of two ellipsoids were constrained in this study. In addition, for consistency with the theoretical considerations, friction was neglected during the compression step in the DEM. Normal and tangential forces were obtained from the history output of interaction in PFC.

**Table 1:** Parameters used in the simulations.

Symbol	Description	Value	Range
E	Young’s modulus	10 GPa	[0,∞)
υ	Poisson ratio	0.3	(0,0.5)
ρ	Density	2500 kg/m^3^	[0,∞)
$\beta_{n}$	Normal critical damping ratio	0.02	[0,1]
$\beta_{s}$	Shear critical damping ratio	0.02	[0,1]
μ	Friction coefficient	0.3	[0,1]
${\dot{\delta }}_{n}$	Relative normal translational velocity	1.0 m/s	[0,∞)
${\dot{\delta }}_{s}$	Relative shear translational velocity	0.5 m/s	[0,∞)

### 3.2. Normal contact force

**Fig 3:**
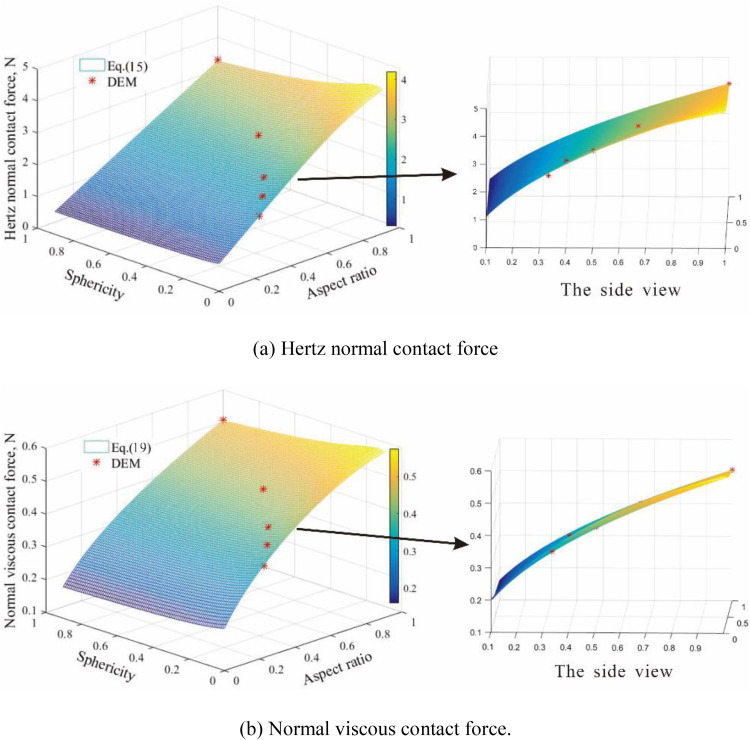
The normal force between two particles with De = 6.30 mm for different aspect ratios and sphericities: (a) hertz normal contact force; (b) normal viscous contact force.

**Fig 4:**
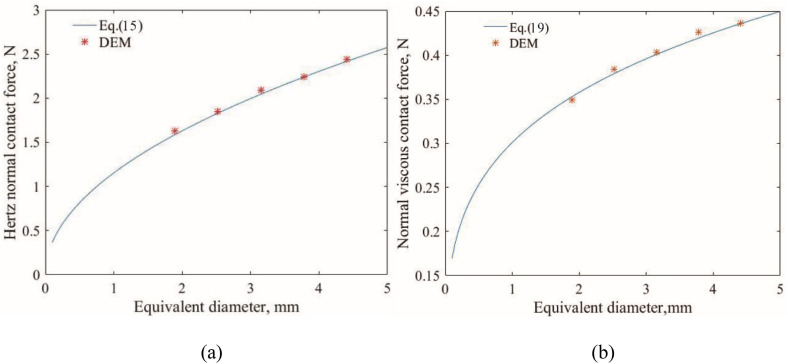
The normal force between two particles with different equivalent diameters for P3: (a) hertznormal contact force; (b) normal viscous contact force.

The normal force between two particles with different aspect ratios and sphericities are shown in Fig 3. Additionally, the theoretical formula Eq.15 and Eq.19 were also plotted in these figures. There is a notable agreement between the theoretical results and DEM results. Fig 3a illustrates the correlation between Hertz normal contact force, aspect ratio and sphericity. The normal contact force gradually increases with the increase in aspect ratio and sphericity. Fig 3b displays the correlations between viscous normal contact force and particle morphology parameters (aspect ratio, and sphericity). Both viscous normal contact force and Hertz normal contact force have similar tendency, increasing as the aspect ratio and sphericity increase. Moreover, the viscous normal contact force is significantly lower than the Hertz normal contact force. Focusing on an ellipsoid (P3) with particle morphology parameters (*Ω* = 0.50, *S*= 0.25), the relationships between equivalent diameters and Hertz normal contact force as well as viscous normal contact force are shown in Fig 4a-b. Both Hertz normal contact force and viscous normal contact force grow with the increasing equivalent diameter, but their rates of increase decrease as the equivalent diameter increases.

### 3.3. Tangential contact force

**Fig 5:**
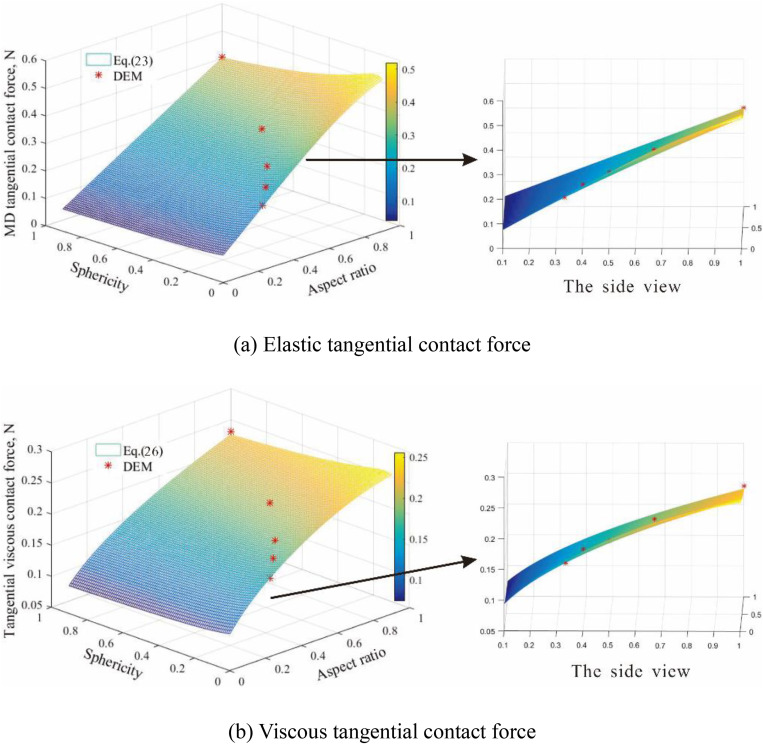
The tangential force between two particles with different aspect ratios and sphericities for De = 6.30 mm: (a) elastic tangential contact force; (b) viscous tangential contact force.

**Fig 6:**
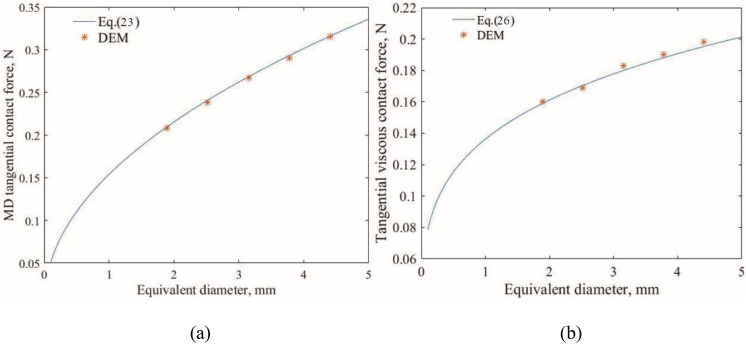
The tangential force between two particles with different equivalent diameters for P3: (a) elastic tangential contact force; (b) viscous tangential contact force.

The correlations of aspect ratio, sphericity, and tangential contact force are demonstrated in Fig 5. There is a good agreement between theoretical results and DEM results. Both tangential elastic contact force and viscous contact force increase as the sphericity and aspect ratio increase. In addition, the tangential elastic contact force is larger than their respective viscous counterparts. Fig 6 describes the correlations between particle equivalent diameters and tangential elastic contact force, as well as viscous contact force. A good agreement between the DEM and theoretical results is evident. Both tangential elastic contact force and viscous contact force increase with the increasing particle equivalent diameter. The numerical results from the two-particle tests showed excellent agreement with the theoretical predictions. The relative error between the DEM-calculated contact forces and the theoretical values was consistently within 5% for all configurations tested, thereby validating the implementation and accuracy of the modified Hertz contact model at the particle scale.

To further validate the discrete element modeling approach and the implementation in PFC5.0, the macroscopic responses predicted by our simulations were compared against established experimental findings in the literature. The DEM parameters and boundary conditions used in this study are consistent with those widely adopted and validated in prior DEM research [42,43], ensuring the reliability of our numerical results.

## 4. Triaxial test simulation

### 4.1. The generation of numerical samples

**Fig 7:**
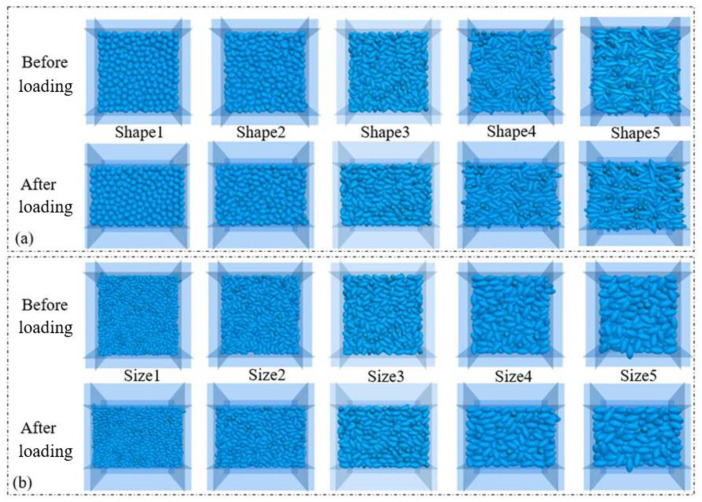
Numerical samples composed of elongated ellipsoids before and after triaxial compression. (a) Samples with different particle morphologies (Shape1-Shape5); (b) Samples with different particle sizes (Size1-Size5).

To investigate the effect of the particle morphology and size on macroscopic mechanical behavior, a series of conventional drained triaxial tests were simulated using DEM. Five numerical samples (Shape1–Shape5) were generated using the following procedure, as illustrated in Fig 7a, each composed of elongated ellipsoids with distinct aspect ratios and sphericities (as defined in Section 3.1) but identical volume, equivalent to a sphere of diameter D = 6.3 mm. Additionally, five samples (Size1–Size5) were created using Particle P3 (see Fig 2) with varying equivalent diameters to isolate the influence of particle size, shown in Fig 7b.

The sample generation process consisted of three main stages. 1) Particle creation and clump modeling: As mentioned in Section 3.1, individual ellipsoidal particles were modeled using the “clump” command in PFC5.0, which rigidly binds overlapping spheres. The bubble packing algorithm [39] was employed to generate the clump templates for each ellipsoid shape (P1-P5), ensuring an accurate geometric representation. 2) Initial specimen generation: These clumps were then randomly generated within a cubic periodic boundary domain measuring 70 mm × 70 mm × 70 mm. The initial placement was performed under a low friction coefficient (e.g., µ = 0.1) to facilitate dense packing. The target initial porosity for all samples was set to approximately 0.25, a value commonly used for dense granular assemblies [42–44]. 3) Equilibration and isotropic compression: Particle generation and equilibration were performed under controlled conditions. Under a confining stress, the assembly was cycled to reach mechanical equilibrium. The system was considered equilibrated when the average unbalanced force ratio fell below the critical threshold of 10 ⁻ ⁵. This ensured that the initial sample was in a stable stress state before shearing. A snapshot of the generated samples before and after loading is provided in Fig.7.

Subsequently, these equilibrated numerical samples underwent isotropic compression via a servo-controlled mechanism to reach the target confining pressure of 400 kPa [43]. Following isotropic compression, the assemblies underwent vertical compression by displacing the top wall downward and the bottom wall upward at a constant velocity. The friction coefficient between particle–wall contacts were set to zero [45–46] to minimize boundary effects. The compression rate is set to 2 mm/s, such that kinetic energy generated during shearing is negligible [42]. The triaxial compression test process ceased upon reaching an axial strain of 25%. The triaxial tests were conducted under drained, dry conditions, meaning no pore fluid was included in the numerical model. The granular assembly was simulated as a dry particulate system, with mechanical behavior governed solely by interparticle contact forces (normal/tangential forces, friction) and boundary loading. Each triaxial test configuration (defined by specific particle morphology and size parameters) was conducted in triplicate (3 independent runs), and the following results represent the mean of these three replicate runs. The generated samples before and after shearing are visualized in Fig 7. The post-shear images are particularly important as they reveal the strain localization and failure patterns, which are analyzed in the following sections.

### 4.2. Stress-strain relationship

**Fig 8:**
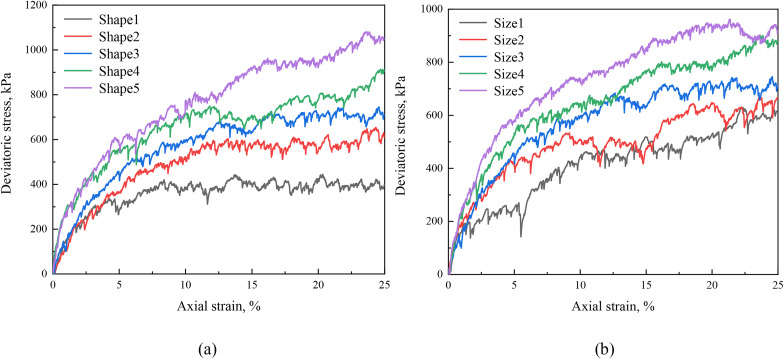
Deviatoric stress-strain curves of numerical samples composed of elongated ellipsoids with different particle morphologies and particle sizes. (a) different particle morphologies; (b) different particle sizes.

Stress-strain relationship is an important macroscopic behavior of samples under triaxial test. The deviatoric stress–axial strain responses under triaxial compression are presented in Fig 8. It shows the influence of particle morphologies and sizes on the deviatoric stress of numerical samples. The relationship between shear strength and particle morphology parameters (aspect ratio and sphericity) is shown in Fig 8a, indicating a significant relationship between particle morphologies and macroscopic mechanical behaviors. Decreases in aspect ratio and sphericity result in increase in both the peak deviatoric stress and deformation modulus, consistent with findings from previous studies [47–52]. This stands in contrast to the particle-scale observations (Sections 3.2 and 3.3), where reduced aspect ratio and sphericity resulted in lower contact forces in two-particle tests. This discrepancy underscores the critical influence of multi-particle interactions and contact-network evolution in assemblies, which cannot be captured by isolated contact scenarios. Additionally, Fig 8b describes the relationship between the particle size and the shear strength, revealing a clear correlation between the two factors. The peak deviatoric stress increases with the increasing particle size, consistent with the results shown in Fig 4 and Fig 6 and the findings in previous studies [53].

### 4.3. Distribution of contact force

**Fig 9:**
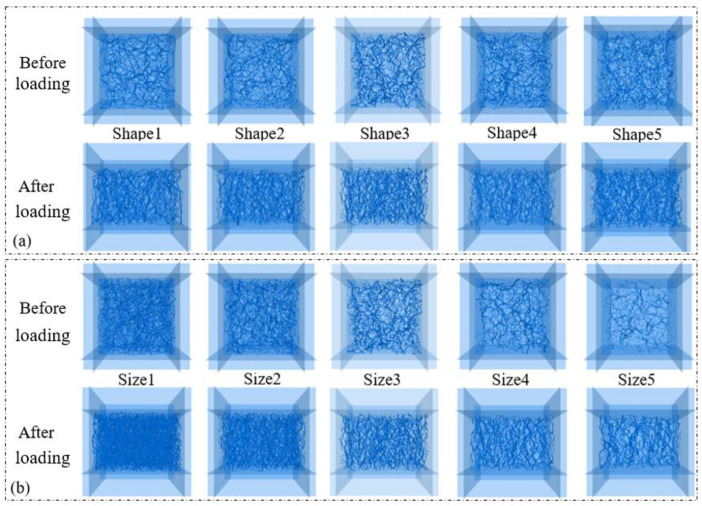
The force chain distributions of different numerical samples composed of elongated ellipsoids with different particle morphologies and particle sizes before shearing and after loading. (a) different particle morphologies; (b) different particle sizes.

**Fig 10:**
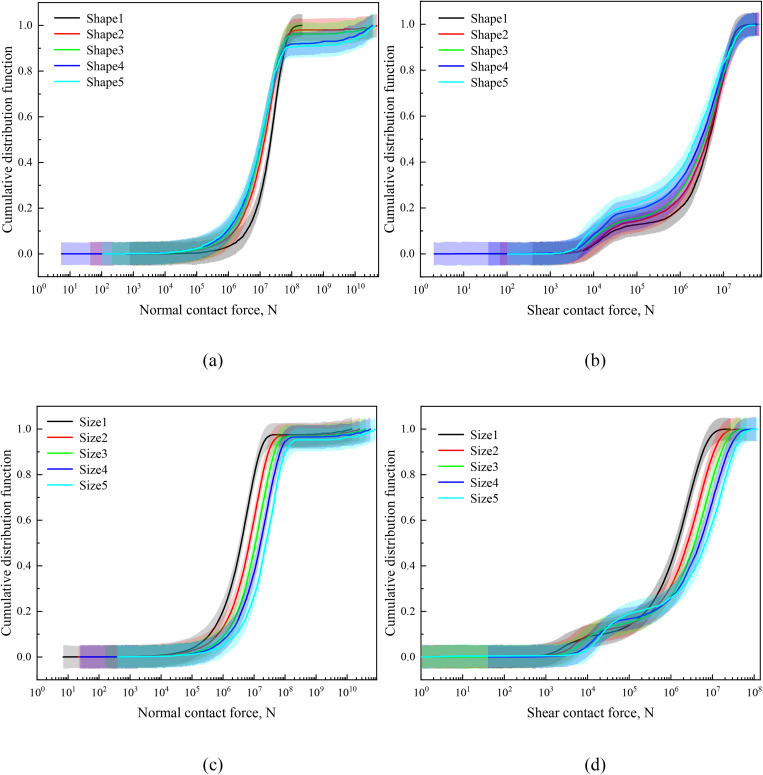
The CDF of normal and tangential contact forces in numerical specimens after compression considering different particle morphologies: (a) normal contact force; (b) tangential contact force; and different particle sizes: (c) normal contact force; (d) tangential contact force.

To elucidate the microscale mechanisms underlying the macroscopic failure behavior, contact force distributions were analyzed. The buckling and reorganization of these force chains represent the micro-scale damage events that collectively lead to macroscopic failure. Force chains are visualized in Fig 9 using segments connecting particle centroids, with line width proportional to force magnitude [21,42]. Before compression, force chains are weak and isotropic. After loading, strong, vertically-aligned force chains develop to carry the major load. The failure of the material is associated with the buckling and collapse of these force chains, which is a dynamic process leading to stress drops in the macroscopic stress-strain curve (Fig 8). Samples with lower sphericity (e.g., Shape5) sustain a more robust and persistent strong force network, which delays the complete failure and leads to higher peak strength. These observations are quantitatively supported by the cumulative distribution functions (CDFs) of normal and tangential contact forces (Fig 10). Notably, Fig 10a and 10c reveal that non-spherical particles exhibit broader distributions of normal contact forces than spherical ones, indicating a greater propensity for stress concentration. Tangential forces (Fig 10b and 10d) show a consistent bimodal distribution, which becomes more pronounced with increasing particle size [51]. This bimodality is likely due to the coexistence of vertically aligned force chains (dominated by compression) and inclined chains (subject to significant shear), as well as the geometric diversity of contacts permitted by non-spherical shapes.

### 4.4. Strong contact force

**Fig 11:**
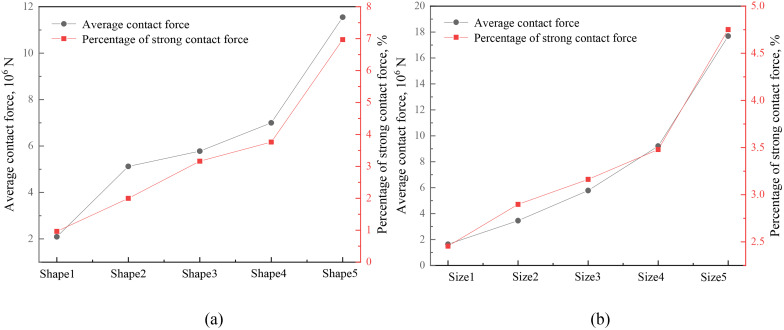
Average contact force and percentage of strong contact force under different conditions: (a) different particle shapes, (b) different particle sizes.

Radjai et al. [54] pointed out average contact force as a criterion for classifying strong and weak contact forces, a method which was verified and used by Liu et al [55]. In this study, contact forces which are more than the average contact force are classified as strong contact forces. Fig 11 shows the variation in average contact force and percentage of strong contact force with different particle shapes and sizes. It can be seen that the average contact force and percentage of strong contact forces increase with the decreasing aspect ratio and sphericity, and increasing equivalent diameter, which can be used to explain the macro mechanical properties in section 4.2 from the particle scale.

### 4.5. Fabric anisotropy

In order to explore further the evolution of contact force orientation, fabric anisotropy values [56] were calculated according to the following equations. The direction of contact can be expressed by a second-order tensor $F_{ij}$.:


$$F_{ij}=\frac{\text{1}}{N}\sum_{k=\text{1}}^{N}n_{i}^{\left(k\right)}n_{j}^{\left(k\right)}\text{i},\text{j}=\text{1},\text{2},\text{3}$$ (27)

where *N* is the total number of contacts, *k* represents the *k*-th contact, and $n_{i}^{\left(k\right)},n_{j}^{\left(k\right)}$ are the normal components of the direction of contact on the *i* and *j* axes, respectively.

**Fig 12:**
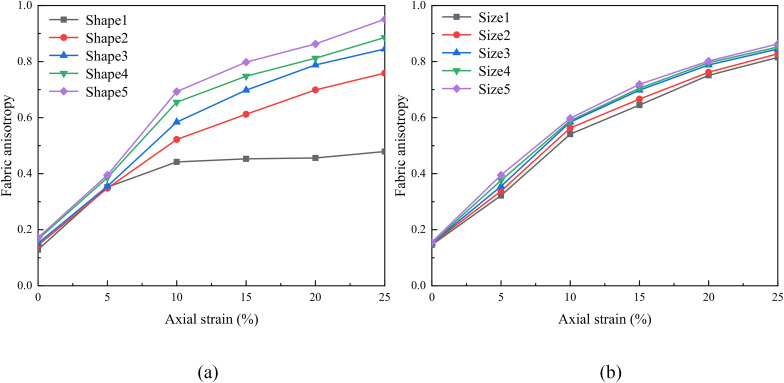
Fabric anisotropy evolution of numerical samples with different shapes and sizes.


This equation includes a single contact arrangement characteristic, and mainly considers the components of the contact direction vector on the 3 orthogonal axes of the group structure. Due to the two-dimensional contact force distribution in Fig 12, it is necessary to adopt two-dimensional graphical analysis, and the two-dimensional constitutive tensor in the x-z plane can be expressed as:

$${\overset{―}{F}}_{ij}=[\begin{array}{cc} {\overset{―}{F}}_{\text{1}\text{1}} & {\overset{―}{F}}_{\text{1}\text{3}}\\ {\overset{―}{F}}_{\text{3}\text{1}} & {\overset{―}{F}}_{\text{3}\text{3}} \end{array}]=\frac{\text{1}}{N}[\begin{array}{cc} \sum_{k=\text{1}}^{N}{\left(\text{sin}\left(\theta^{\left(k\right)}\right)\right)}^{\text{2}} & \sum_{k=\text{1}}^{N}\text{cos}\left(\theta^{\left(k\right)}\right)\text{sin}(\theta^{\left(k\right)})\\ \sum_{k=\text{1}}^{N}\text{cos}\left(\theta^{\left(k\right)}\right)\text{sin}(\theta^{\left(k\right)}) & \sum_{k=\text{1}}^{N}{\left(\text{cos}\left(\theta^{\left(k\right)}\right)\right)}^{\text{2}} \end{array}]$$ (28)

where and $\theta^{\left(k\right)}$ is the angle between the contact vector and the z axis. Then the fabric anisotropy of the fabric tensor $F_{ij}$ can be expressed as follows [56]:


$$\Delta =\sqrt{{\left({\overset{―}{F}}_{\text{1}\text{1}}-{\overset{―}{F}}_{\text{3}\text{3}}\right)}^{\text{2}}+\text{4}{\overset{―}{F}}_{\text{1}\text{3}}{\overset{―}{F}}_{\text{3}\text{1}}}=\frac{\text{1}}{N}\sqrt{{\left(\sum_{k=\text{1}}^{N}cos\text{2}\theta^{\left(k\right)}\right)}^{\text{2}}+{\left(\sum_{k=\text{1}}^{N}sin\text{2}\theta^{\left(k\right)}\right)}^{\text{2}}}$$ (29)

Then the fabric anisotropy values were calculated at axial strains of 0%, 5%, 10%, 15%, 20%, 25% under different confining pressures in Fig 12. The fabric anisotropy value increases as the axial strain increases. And the fabric anisotropy value increase with the decreasing aspect ratio and sphericity, and increasing equivalent diameter, which is consistent with the macro stress-strain relationship in Section 4.2. This confirms that enhanced particle alignment and directional force chains directly contribute to higher peak strength.

### 4.6 Critical state stress ratio

The discrete element simulations in this study can be interpreted within the framework of Critical State Soil Mechanics (CSSM). CSSM provides a unifying framework to describe the stress-strain behavior of soils under shear. The core concept is that all soils, when subjected to continuous shear deformation, will ultimately reach a critical state where they continue to deform without changes in stress [57–58].

The mean effective stress *p* and deviatoric stress *q* are defined as:


$$p=\frac{\left(\sigma_{\text{1}}+{\text{2}\sigma }_{\text{3}}\right)}{\text{3}}$$ (30)

$$q=\sigma_{\text{1}}-\sigma_{\text{3}}$$ (31)

where $\sigma_{\text{1}}$ and $\sigma_{\text{3}}$ are the major and minor effective principal stresses, respectively. At critical state, all stress paths converge to a unique line, the Critical State Line (CSL), in the *p- q* space, which is commonly expressed as:


q=γp (32)

where γ is the critical state stress ratio.

Therefore, the critical state stress ratios of the numerical specimens under different sizes and morphologies can be calculated based on the above content, and the data are summarized in Table 2.

**Table 2:** Critical state parameters obtained from DEM simulations.

Sample Name	Aspect Ratio	Sphericity	Equivalent Diameter (mm)	Critical State Stress Ratio
Shape1	1.00	1.00	6.30	1.25
Shape2	0.67	0.44	6.30	1.38
Shape3	0.50	0.25	6.30	1.45
Shape4	0.40	0.16	6.30	1.52
Shape5	0.33	0.11	6.30	1.60
Size1	0.50	0.25	3.78	1.42
Size2	0.50	0.25	5.04	1.44
Size3	0.50	0.25	6.30	1.45
Size4	0.50	0.25	7.56	1.47
Size5	0.50	0.25	8.82	1.49

The incorporation of the CSSM framework allows for a deeper interpretation of the effects of particle morphology and size. As summarized in Table 2, the critical state stress ratio increases systematically with decreasing aspect ratio and sphericity. This indicates that more elongated and non-spherical particles develop a higher frictional resistance at critical state, which is consistent with their enhanced interlocking and the formation of more stable, anisotropic force chains (as discussed in Sections 4.3 and 4.5).

## 5. Discussion

In the context of this study on cohesionless granular materials, failure behavior refers to the irreversible evolution of the internal granular structure under load. This includes the loss of initial fabric, and the collective buckling and reformation of force chains. Unlike cemented materials, there is no tensile cracking, but the constitutive response is governed by these granular-scale restructuring processes.

### 5.1. Underlying mechanisms for multiscale behavior

**Fig 13:**
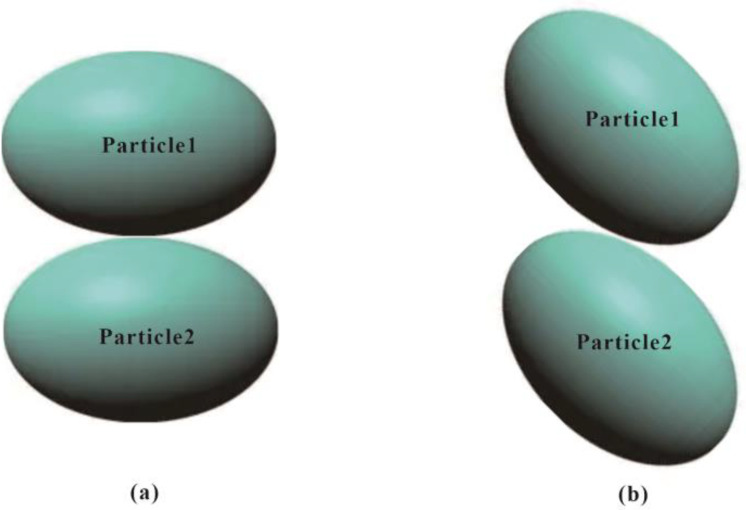
Different contact patterns for two ellipsoids.

The findings of this study reveal a seemingly paradoxical trend: at the particle scale, a decrease in aspect ratio and sphericity leads to a decrease in normal and tangential contact forces for head-on collisions (Section 3). Conversely, at the sample scale, the same morphological change results in a significant increase in peak deviatoric stress and average contact force (Sections 4.2, 4.4). The key is to recognize that the simplified head-on contact (Fig. 13a) represents only one of many possible contact configurations in a dense assembly (Fig 13b). The macroscopic strength is not governed by the average force in idealized contacts, but by the emergence and percolation of a strong force network dominated by highly stressed, interlocking contacts.

In a polydisperse assembly of non-spherical particles under shear, contacts frequently occur at obliquely oriented edges and vertices. At these points, the local principal radii of curvature are significantly smaller than those in a head-on configuration. According to Eqs. 15 and 23, a smaller radius of curvature (*R*_*c*_) generates a larger contact force for the same displacement. Therefore, the increased presence of these “high-stress” contact points in assemblies of more elongated (low *Ω*), non-spherical (low *S*) particles dominates the macroscopic response, leading to higher overall strength and stronger force chains. This explanation is supported by the observed increase in the stress concentration factor *K*_*mo*r_ (Table 3) and the proportion of strong contacts (Fig 11) with decreasing sphericity and aspect ratio. $K_{mor}$ was calculated as the ratio of the local maximum contact stress $\sigma_{max}$ to the average contact stress $\overset{―}{\sigma }$ [59,60].

**Table 3:** Stress concentration factor value in the numerical samples with different shapes and sizes.

	Shape1	Shape2	Shape3	Shape4	Shape5
$K_{mor}$	2.63	5.24	7.81	10.33	18.48
	Size1	Size2	Size3	Size4	Size5
$K_{mor}$	4.71	5.86	7.81	9.71	11.00


$$K_{mor}=\frac{\sigma_{max}}{\overset{―}{\sigma }}=\frac{{\left\langle A_{c}\right\rangle \sigma }_{max}}{\left\langle F_{c}\right\rangle }$$ (33)

where $\left\langle A_{c}\right\rangle$ is the average contact area, and $\left\langle F_{c}\right\rangle$ is the average contact force.

As shown in Table 3, $K_{mor}$ increases dramatically from 2.63 for spheres (Shape1) to 18.48 for the most elongated particles (Shape5)—a 600% increase. This demonstrates a fundamental shift in the contact force distribution, where a larger proportion of the applied load is carried by a smaller number of highly stressed contacts. The increased stress concentration directly feeds the development of a more robust strong contact network. Fig 11a shows that the proportion of strong contacts (those carrying above-average force) increases from approximately 40% in Shape1 (spheres) to over 60% in Shape5—a relative increase of 50%. Although an individual complex contact might be theoretically weaker than an ideal head-on one, the collective network of these interlocking, stress-concentrating contacts is far more efficient at transmitting load and resisting shear. This network is characterized by stronger fabric anisotropy (Fig 12), which directly contributes to higher peak strength.

The integration of local stress concentration ($K_{mor}$) across multiple contacts to yield a net increase in bulk shear strength is a statistical process governed by two key factors: the percolation of strong forces and the fabric anisotropy of the contact network. A high $K_{mor}$ value signifies that a significant portion of the external load is borne by a relatively small subset of contacts. As shown in Fig 11a, the increased $K_{mor}$ in non-spherical assemblies is statistically coupled with a higher percentage of strong contacts. These strong contacts do not act in isolation; they connect to form a system-spanning “strong force network” that percolates through the entire sample [54]. Additionally, the contribution of a contact force to the macroscopic deviatoric stress is maximized when its orientation is aligned with the compression direction. As demonstrated in Fig 12, the fabric anisotropy increases more significantly in non-spherical particle assemblies during shearing. This indicates that the strong contacts in these assemblies are not only more numerous but also more effectively oriented to resist the applied shear.

### 5.2. The origin of bimodal distribution in tangential forces

The observed bimodal distribution in tangential contact forces (Fig 10b, d) is a noteworthy microscopic finding. This phenomenon likely stems from two interrelated mechanisms during triaxial compression. First, the development of anisotropic force chains (Fig 9) creates distinct populations of contacts: (i) those in vertically-oriented chains, primarily carrying compressive loads with limited relative slip, and (ii) those in inclined or transverse chains, which experience significant shear components and micro-slip. These two populations naturally develop different ranges of tangential force magnitudes, manifesting as two peaks in the cumulative distribution function.

Second, the diversity of contact configurations between non-spherical particles plays a critical role. Due to their elongated morphology, ellipsoids can interact through various geometric configurations—from near-frontal contacts (larger *R*_*c*_, smaller tangential force) to contacts near the particle tips (smaller *R*_*c*_, larger tangential force). This spectrum of contact geometries directly results in a broader distribution of tangential forces, which under the constrained conditions of shear deformation, evolves into a bimodal distribution. This finding aligns with the concepts of strong force networks discussed by Radjai et al. [54] and adds granularity to their model by linking force heterogeneity directly to particle morphology.

### 5.3. Comparison with previous studies and novel insights

Our results at the sample scale confirm well-established trends in the literature: that more angular and elongated particles lead to higher shear strength and dilatancy [21,47,49]. To address the validation at the sample scale, the macroscopic behavior predicted by our DEM model was compared with experimental observations from the literature. For instance, Yang & Luo [18] experimentally demonstrated that assemblages of more angular and elongated particles exhibit significantly higher peak shear strength and more pronounced dilatancy compared to rounded particles. As shown in Fig 8a of our study, our simulations consistently replicate this trend: a decrease in sphericity (S) from 1.00 to 0.11 leads to a 15–40% increase in peak deviatoric stress, which is in good agreement with the 20–50% enhancement reported in their experimental work on sands with similar shape variations. Furthermore, the observed increase in strength with particle size (Fig 8b) aligns with the experimental results on rockfill materials reported by Han et al. [53]. This qualitative and quantitative agreement with independent experimental data provides strong support for the overall validity of our modified contact model and the DEM framework in capturing the essential mechanics of granular materials influenced by particle morphology and size.

However, our study moves beyond these macroscopic correlations by providing a mechanistic, multiscale explanation. While previous DEM studies [21,42,47] often attributed the increased strength of non-spherical particles solely to interlocking and reduced rotation, our analysis quantitatively links it to the fundamental mechanics of contact and the ensuing stress concentration. The introduction of the stress concentration factor *K*_*mor*_ provides a novel metric to quantify this effect. A higher *K*_*mor*_ value indicates a greater propensity for stress localization, which not only enhances macroscopic friction but also predicts potential locations for particle crushing—a phenomenon of great importance in geotechnics [16,17]. This provides a missing link between particle-scale mechanics and bulk performance.

### 5.4. Implications of the multiscale framework

The core novelty of this work lies in its integrated multiscale framework. By employing the same morphological parameters (*Ω*, *S*, *D*_*e*_) in both the particle-scale contact model and the sample-scale simulations, we establish a direct and consistent causal chain. This approach resolves the ambiguity often encountered in single-scale studies. We demonstrate that the influence of particle morphology is not monolithic but is scale-dependent and pattern-dependent: while head-on contacts show one trend, the ensemble average of all contacts in an assembly (dominated by complex configurations) reveals the opposite and governing trend.

This framework is not merely descriptive but possesses predictive power. The validated modified Hertz model allows for the estimation of contact forces based on particle shape and size, which can be used to inform larger-scale simulations or interpret experimental results. For engineering practice, this implies that tailoring particle morphology—for instance, in designing backfill materials or asphalt mixes—can be approached more scientifically. Selecting particles with specific shapes can be targeted to either enhance strength (through higher stress concentration and interlocking) or improve compressibility (using more rounded particles).

### 5.5. Comparative analysis and model performance

To quantitatively evaluate the improvements offered by our modified contact model, a comparative analysis was conducted against the classical Hertz-Mindlin model for spheres and other relevant studies from the literature. The comparison, summarized in Table 4, spans both particle-scale and sample-scale performance.

**Table 4:** Comparative analysis of the proposed modified contact model against previous approaches.

Aspect of Comparison	Classical Hertz-Mindlin (Spheres)	Representative Previous Studies [21–22]	This Study (Proposed Model)
Morphology Description	None (Perfect spheres)	Often uses multi-sphere clumps; morphology is geometric but not explicitly linked to *R*ₘ in contact law.	Explicit parameters (Ω, S, Dₑ) directly integrated into the contact theory via *R*ₘ (Ω,S,Dₑ,θ).
Macroscopic Stress-Strain Prediction	Fails to capture strength increase with non-sphericity.	Can capture trends but often through geometric interlocking alone.	Captures strength increase matching experimental trends, Provides micro-mechanical explanation.
Micro-Mechanical Insight	Limited to spherical packings.	Focus on fabric, coordination number.	Direct link from morphology to contact force and stress concentration, explaining macro-behavior at its origin.

## 6. Conclusions

The multiscale analysis of contact forces for particles with various particle morphologies and sizes was conducted using contact mechanics theory and DEM. The modified Hertz contact model was established and validated through two-particle tests and triaxial simulations. The main conclusion can be summarized as follows:

(1) The modified Hertz contact model demonstrated high accuracy, with numerical results showing strong agreement with theoretical predictions. This model effectively captures the effects of particle morphology and size on mechanical behavior at the particle scale.
(2) Decreases in aspect ratio (from 1.00 to 0.33) and sphericity (from 1.00 to 0.11) resulted in a 30–50% increase in average contact force and a 15–25% increase in the proportion of strong contact forces. Similarly, increasing the equivalent diameter from 3.78 mm to 8.82 mm led to a 40–60% rise in average contact force. These particle-scale trends are consistent with sample-scale observations, where the same morphological and size changes caused a 15–40% increase in peak deviatoric stress.
(3) The study provides a quantitative multiscale framework linking particle characteristics to macroscopic mechanical behavior. The results offer practical insights for optimizing material design in geotechnical and particulate systems, such as tailoring particle shape and size distribution to enhance shear strength and stability.

### Permits

All necessary permits were obtained for the described study, which complied with all relevant regulations.

## Appendix A

### Calculation of the principal curvatures

As mentioned above, the ellipsoid is represented by the following equation,


$$\frac{x^{\text{2}}}{a^{\text{2}}}+\frac{y^{\text{2}}}{b^{\text{2}}}+\frac{z^{\text{2}}}{c^{\text{2}}}=\text{1}(a\leq b\leq c)$$ (A1)

Solving for z:


$$z=c\sqrt{\text{1}-\frac{x^{\text{2}}}{a^{\text{2}}}-\frac{y^{\text{2}}}{b^{\text{2}}}}$$ (A2)

$$z=z(x,y)=(x,y,c\sqrt{\text{1}-\frac{x^{\text{2}}}{a^{\text{2}}}-\frac{y^{\text{2}}}{b^{\text{2}}}})$$ (A3)

Suppose the contact point P is $z(x_{\text{0}},y_{\text{0}})$, then a differential vector at point P can be written as:


$$dz=z\left(x_{\text{0}}+dx,y_{\text{0}}+dy\right)-z(x_{\text{0}},y_{\text{0}})$$ (A4)

It can be also written in another form:


$$dz=\frac{\partial z}{\partial x}dx+\frac{\partial z}{\partial y}dy$$ (A5)

The dot product of dz is


$$dz\cdot dz=\frac{\partial z}{\partial x}\cdot \frac{\partial z}{\partial x}dx^{\text{2}}+\frac{\partial z}{\partial x}\cdot \frac{\partial z}{\partial y}dxdy+\frac{\partial z}{\partial y}\cdot \frac{\partial z}{\partial y}dy^{\text{2}}$$ (A6)

is called the ‘first fundamental form’ of the surface at the point P. The ‘first fundamental coefficients’ E, F and G are the abbreviation of the coefficients in Equation.


$$E=\frac{c^{\text{4}}x^{\text{2}}}{a^{\text{4}}z^{\text{2}}}+\text{1}F=\frac{c^{\text{4}}xy}{a^{\text{2}}b^{\text{2}}z^{\text{2}}}G=\frac{c^{\text{4}}y^{\text{2}}}{b^{\text{4}}z^{\text{2}}}+\text{1}$$ (A7)

A vector normal to the surface at P is defined the cross product:


$$n=\frac{\partial z}{\partial x}\times \frac{\partial z}{\partial y}$$ (A8)

The length of the normal vector is:

$$n=\left|\frac{\partial z}{\partial x}\times \frac{\partial z}{\partial y}\right|\text{and}\hat{n}=\frac{n}{n}$$ (A9)

The ‘second fundamental form’ of the surface at P can be expressed as:


$$dz\cdot dz\cdot \hat{n}=\frac{\partial z}{\partial x}\cdot \frac{\partial z}{\partial x}\cdot \hat{n}dx^{\text{2}}+\frac{\partial z}{\partial x}\cdot \frac{\partial z}{\partial y}\cdot \hat{n}dxdy+\frac{\partial z}{\partial y}\cdot \frac{\partial z}{\partial y}\cdot \hat{n}dy^{\text{2}}$$ (A10)

The ‘second fundamental coefficients’ L, M and N are the abbreviation of the coefficients in Equation.


$$L=\frac{c^{\text{4}}{(b}^{\text{2}}-y^{\text{2}})}{a^{\text{2}}b^{\text{2}}{hz}^{\text{3}}}M=\frac{c^{\text{4}}xy}{a^{\text{2}}b^{\text{2}}{hz}^{\text{3}}}N=\frac{c^{\text{4}}{(a}^{\text{2}}-x^{\text{2}})}{a^{\text{2}}b^{\text{2}}{hz}^{\text{3}}}h=\sqrt{\text{1}+\frac{c^{\text{4}}x^{\text{2}}}{a^{\text{4}}z^{\text{2}}}+\frac{c^{\text{4}}y^{\text{2}}}{b^{\text{4}}z^{\text{2}}}}$$ (A11)

The curvature of the surface at point P


$$\kappa =\frac{Ldx^{\text{2}}+\text{2}Mdxdy+Ndy^{\text{2}}}{Edx^{\text{2}}+\text{2}Fdxdy+Gdy^{\text{2}}}$$ (A12)

Angle θ is defined such that:


$$\text{tan}\theta =\frac{dy}{dx}$$ (A13)

The range of θ is that −π/2≤θ≤π/2 for dx≠0; when dx=0, θ=π/2.


$$\frac{\text{1}}{R_{c}}=\kappa =\frac{L\;{\text{cos}}^{\text{2}}\;\theta +\text{2}M\;\text{sin}\;\theta \;\text{cos}\;\theta +N\;{\text{sin}}^{\text{2}}\;\theta }{E\;{\text{cos}}^{\text{2}}\;\theta +\text{2}F\;\text{sin}\;\theta \;\text{cos}\;\theta +G\;{\text{sin}}^{\text{2}}\;\theta }$$ (A14)

Where $R_{c}$ is the principal radii of curvatures.

Then $R_{c}$ can be described by particle morphology and size parameters like the following equation.


$$R_{c}=R_{c}(\Omega ,S,D_{e})$$ (A15)

At a point on a surface the curvature usually depends on direction θ. The extrema of curvature κ over θ are the two principal curvatures at the point and the corresponding angles θ define the two corresponding principal directions. Note that the principal directions are orthogonal and given by the solutions to the equation.


$$(EM-FL)\;{\text{cos}}^{\text{2}}\;\theta +(EN-GL)\;\text{sin}\;\theta \;\text{cos}\;\theta +(FN-GM)\;{\text{sin}}^{\text{2}}\;\theta =\text{0}$$ (A16)
